# Dedicated Echoendoscope for Interventional Endoscopic Ultrasound: Comparison with a Conventional Echoendoscope

**DOI:** 10.3390/jcm13102840

**Published:** 2024-05-11

**Authors:** Toshio Fujisawa, Shigeto Ishii, Yousuke Nakai, Hirofumi Kogure, Ko Tomishima, Yusuke Takasaki, Koichi Ito, Sho Takahashi, Akinori Suzuki, Hiroyuki Isayama

**Affiliations:** 1 Department of Gastroenterology, Graduate School of Medicine, Juntendo University, 2-1-1 Hongo, Bunkyo-ku, Tokyo 113-8421, Japan; t-fujisawa@juntendo.ac.jp (T.F.); sishii@juntendo.ac.jp (S.I.); tomishim@juntendo.ac.jp (K.T.); ytakasa@juntendo.ac.jp (Y.T.); kitoh@juntendo.ac.jp (K.I.); sho-takahashi@juntendo.ac.jp (S.T.); suzukia@juntendo.ac.jp (A.S.); 2 Department of Gastroenterology, Graduate School of Medicine, The University of Tokyo, Tokyo 113-8655, Japan; 3 Department of Internal Medicine, Institute of Gastroenterology, Tokyo Women’s Medical University, Tokyo 162-8666, Japan; 4 Division of Gastroenterology and Hepatology, Department of Medicine, Nihon University School of Medicine, Tokyo 173-8610, Japan; kogure.hirofumi@nihon-u.ac.jp

**Keywords:** endoscopic ultrasound (EUS), interventional EUS, EUS-guided biliary drainage (EUS-BD), fluoroscopy time, adverse events

## Abstract

**Background/Objective**: Interventional endoscopic ultrasound (I-EUS) is technically difficult and has risks of severe adverse events due to the scarcity of dedicated endoscopes and tools. A new EUS scope was developed for I-EUS and was modified to increase the puncture range, reduce the blind area, and overcome guidewire difficulties. We evaluated the usefulness and safety of a new EUS scope compared to a conventional EUS scope. **Methods**: All I-EUS procedures were performed at Juntendo University Hospital from April 2020 to April 2022. The primary outcomes included the procedure time and fluoroscopy time. The secondary outcomes included the technical success rate and the rates of procedure-related adverse events. Clinical data were retrospectively reviewed and statistically analyzed between the new and conventional EUS scopes. **Results**: In total, 143 procedures in 120 patients were analyzed. The procedure time was significantly shorter with the new EUS scope, but the fluoroscopy time was not different. Among the patients only undergoing EUS-guided biliary drainage (EUS-BD), 79 procedures in 74 patients were analyzed. Both the procedure time and fluoroscopy time were significantly shorter with the new EUS scope. Multivariate analysis revealed that a new EUS scope and use of covered metal stents could reduce the fluoroscopy time. The technical success rate and the adverse event rate were not significantly different between the total I-EUS and the EUS-BD only groups. However, the conventional scope showed stent deviation during stent placement, which did not happen with the new scope. **Conclusions**: The new EUS scope reduced procedure time for total I-EUS and fluoroscopy time for EUS-BD compared to a conventional EUS scope because of the improvement suitable for I-EUS.

## 1. Introduction

Procedures that involve endoscopic ultrasound (EUS) are frequently performed worldwide [1,2,3]. Diagnostic EUS and EUS-guided fine needle aspiration are basic procedures at academic and referral centers, while an interventional EUS (I-EUS) is technically difficult and has a risk of serious adverse events [4,5]. Specifically, EUS-guided drainage/anastomosis (EUS-D/A) of the bile duct, gallbladder, pancreatic duct, and digestive tract are technically challenging procedures [6,7,8]. As such, they are only conducted at facilities where there are experts with a wealth of experience in I-EUS. In addition, the lack of dedicated endoscopes and devices for I-EUS makes the procedure difficult and hinders the standardization of I-EUS [9,10].

A new EUS scope was developed to improve the I-EUS process [11]. The current scopes are difficult to perform I-EUS because they were developed for a diagnostic EUS and EUS-guided tissue acquisition. There are several reasons for this. First, the inability of the endoscope to make small turns and the poor mobility of the puncture needle limit the areas where the target can be punctured. This restricts the bile duct branches that can be punctured, hampering subsequent procedures, and increasing the risk of esophageal puncture [12]. Second, there is a blind spot for puncturing because of the distance between the ultrasound transducer and the accessory channel. This increases the probability of double puncture of the mucosa and unintended puncture of a blood vessel [13,14]. Third, it is difficult to visualize stent release and see a deployed stent in the endoscopic view, which can lead to stent misplacement [15,16]. Fourth, there is no guidewire (GW) locking system, so there is a risk of the GW slipping or falling off during device replacement, necessitating replacement under fluoroscopy [17]. This results in longer radiation exposure time and procedure time. The new EUS scope, EG-740UT (Fujifilm Corporation, Tokyo, Japan), has features to overcome these issues.

We evaluated the new scope for I-EUS. We focused on the fluoroscopy time, which is directly linked to health problems among operators and patients, and compared the results to those using a conventional EUS scope (EG-580UT, Fujifilm Corporation).

## 2. Methods

### 2.1. Study Design

This retrospective observational study compared the efficacy and safety of I-EUS using the two scopes mentioned above. The study was approved by the Institutional Review Board of Juntendo University Hospital (E22–0378, approved on 1 December 2022) and was conducted in accordance with the principles of the Declaration of Helsinki.

### 2.2. Inclusion and Exclusion Criteria

We reviewed the data of all patients who underwent I-EUS at Juntendo University Hospital, Tokyo, Japan, from April 2020 to April 2022. All patients who underwent I-EUS were included in the initial analysis. The procedures included EUS-guided biliary drainage (EUS-BD), EUS-guided pancreatic duct drainage (PDD), EUS-guided peripancreatic fluid drainage (EUS-PFD), the EUS-assisted rendezvous technique, and EUS-guided gastrojejunostomy. The inclusion criteria were obstructive jaundice and/or cholangitis due to malignant or benign disease, and drainage from the intrahepatic bile duct including EUS-guided hepaticogastrostomy (EUS-HGS) or EUS-guided hepaticojejunostomy (EUS-HJS) after total gastrectomy. The exclusion criterion was drainage from the extrahepatic bile duct, including EUS–choledochoduodenectomy (EUS-CDS). 

First, the entire I-EUS was analyzed (entire cohort). However, for an entire I-EUS, the analysis included subjects with heterogeneous patient characteristics and procedures. Therefore, the analysis was limited to EUS-BD, the main technique of I-EUS, as the second analysis (limited cohort).

### 2.3. Improvements of the New EUS Scope (Table 1)

**Figure 1:**
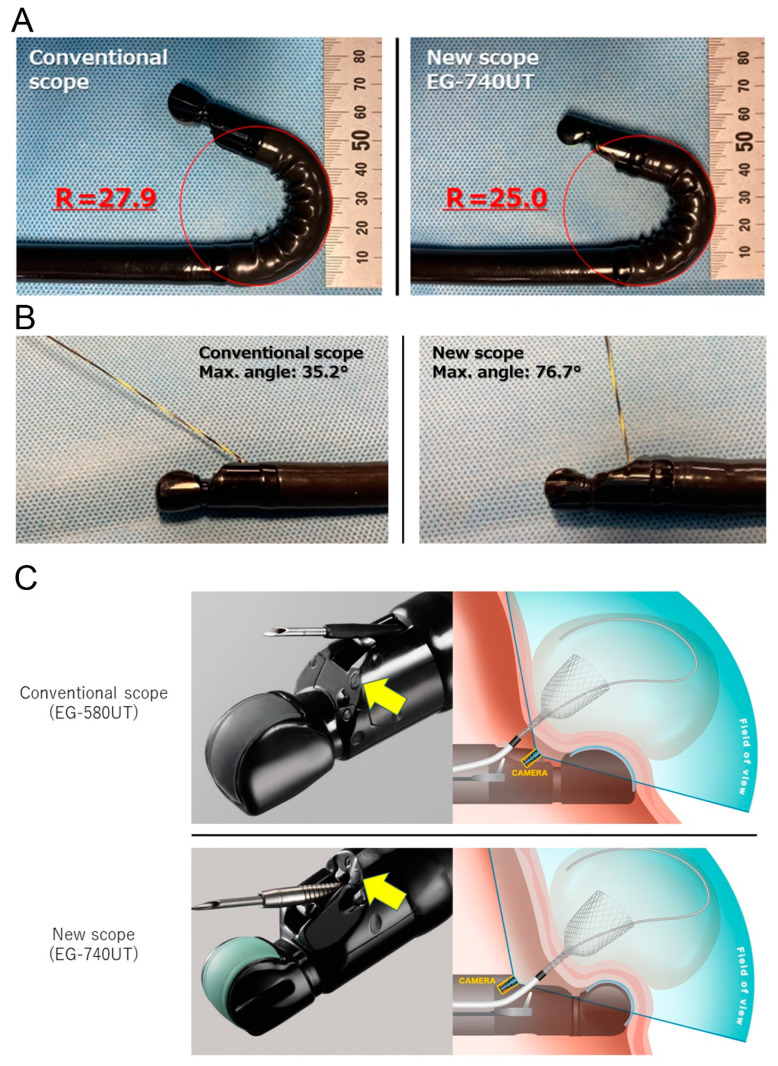
Performance of a conventional scope (EG-580 UT) and the new scope (EG-740 UT). (**A**) The radius of curvature of the flexible tip of the scope was decreased from 27.9° to 25.0°. This improvement made the scope more nimble. (**B**) The strength of the forceps elevator was increased. In an experiment using a 0.025-inch guidewire, the elevation angle increased from 35.2° to 76.7°. (**C**). The CCD camera (yellow arrows) was behind the forceps channel in the new scope. Stent deployment was easily observed.

The scanning area was increased from 150° to 180°, reducing the blind area and preventing an unintended puncture of a blood vessel. The radius of curvature of the flexible tip was decreased from 27.9° to 25.0° (Figure 1A), which made the scope nimble and expanded the potential puncture area. The strength of the forceps elevator was increased. In an experiment in vitro using a 0.025-inch guidewire, the maximum elevation angle increased from 35.2° to 76.7° (Figure 1B). This improvement widened the puncture area, making the procedures safer. The GW locking system holds the GW tightly, preventing slippage during device exchange. With the GW locking system, devices can be replaced without checking the GW position under fluoroscopy. The charge-coupled device (CCD) lens was repositioned behind the working channel, enabling it to easily deploy the stent while viewing with endoscopy and reducing the blind area by shortening the distance between the transducer and the working channel (Figure 1C).

**Table 1:** Improvement points of the newly developed EUS scope.

	Mechanism	Improvement Point	Expected Effect
1	Scanning area under ultrasonography	Widen from 150° to 180°	Reduce blind area
2	Radius of curvature of the flexible tip	Decrease from 27.9° to 25.0°	Widen puncture area
3	Forceps elevator	Increase elevating angle of the elevator	Widen puncture area
4	Guidewire locking system	Holding the guidewire tightly	Shorten device exchange time
5	CCD camera	Moving CCD behind the working channel	Reduce stent misdeployment

CCD: charge coupled device.

### 2.4. I-EUS

I-EUS was performed using both conventional and new EUS scopes, and SU-1 was used as the ultrasonography processor. The conventional EUS scope was mainly used before the launch of the new EUS scope (April 2020 to March 2021), and the new EUS scope was used thereafter (April 2021 to April 2022). A method of EUS-HGS, the main procedure of EUS-BD, is presented. A standard 19-gauge needle was used to puncture the left intrahepatic bile duct under EUS guidance in a color doppler mode to avoid intervening blood vessels. A 22-gauge needle was used if the targeted bile duct was not sufficiently dilated [18]. The B3 branch was preferred as the puncture site over the B2 branch because a B2 puncture can cause a transesophageal puncture, leading to mediastinitis. A contrast medium was injected into the bile duct, and a 0.025-inch guidewire (Visiglide2, Olympus Medical Systems) was advanced. The puncture tract was initially dilated using an ultra-tapered bougie dilator (ES Dilator, Zeon Medical, Tokyo, Japan) followed by a balloon dilator (REN, Kaneka Medix, Tokyo, Japan). In the case a 22-gauge needle had to be used because some of the intrahepatic bile ducts were encountered, a 0.018-inch guidewire (Fielder, Asahi Intech., Tokyo, Japan) was employed. After the puncture tract was dilated, it was exchanged for a 0.025-inch guidewire. Finally, plastic or metallic stents were placed across the puncture-created route. Metallic stents were primarily used for patients with malignant diseases, and plastic stents were used for those with benign diseases. We used the double-GW method, in which two GWs were placed before stenting, to ensure the stability and safety of the plastic stent placement [19,20]. Fluoroscopy was controlled by an assistant inside the procedure room. Adverse events and stent placement were routinely checked by computed tomography (CT) one day after the procedure [21].

### 2.5. Study Outcomes

The primary outcomes included the procedure time and fluoroscopy time of the procedures in both the first (entire cohort) and the second (limited cohort) analysis. The secondary outcomes included the rates of the technical success and the procedure-related AEs, such as stent migration, peritonitis, biloma, bleeding, cholangitis, pancreatitis, and perforation. Clinical success was not an outcome because it varied depending on the procedure.

## 3. Definitions

Technical success was defined as completion of the procedure, such as stent placement, and achievement of the objective. The procedure time was defined as the duration from the insertion of the endoscope to its removal. An expert is defined as an operator that has conducted at least 50 I-EUS procedures and can complete the procedure without an instructor, and a trainee is defined as an operator who does not meet the requirements. Three experts (HI, TF, and SI) and five trainees performed the procedures. The severity of comorbidities in the included patients was assessed using the Charlson comorbidity index [22,23]. The AEs were graded according to the severity grading system of the American Society for Gastrointestinal Endoscopy lexicon [24]. Biliary peritonitis was defined as a condition with symptoms such as abdominal pain and fever but no fluid collection in CT. Biloma was defined as new fluid collection around the puncture site or in the abdominal cavity in CT with or without symptoms. Pancreatitis was defined as an increase in pancreatic enzymes accompanied by symptoms such as abdominal pain. An increase in pancreatic enzymes alone was not considered an AE.

## 4. Statistical Analysis

Statistical analyses were performed to identify significant differences between the new and conventional EUS scopes in terms of patient characteristics, procedural factors, and procedural results. Categorical variables were analyzed using a chi-square test or Fisher’s exact test. Continuous variables are expressed as the medians with interquartile ranges (IQRs) and were analyzed using the Mann–Whitney U test. Multivariate analysis was performed using binominal logistic regression analysis. Patient information (age, sex, and primary diseases [benign or malignant]) and procedure information (procedure type, puncture site, stent type, technical success, procedure time, fluoroscopic time, and operator experience) were retrospectively retrieved from the medical records and tabulated using Excel software (v. microsoft 365, Microsoft Corp, Redmond, WA). Statistical significance was set at *p* < 0.05. Statistical analysis was performed using SPSS (v. 24; IBM SPSS, Chicago, IL, USA) [25].

## 5. Results

### 5.1. Total I-EUS (First Examination)

**Figure 2:**
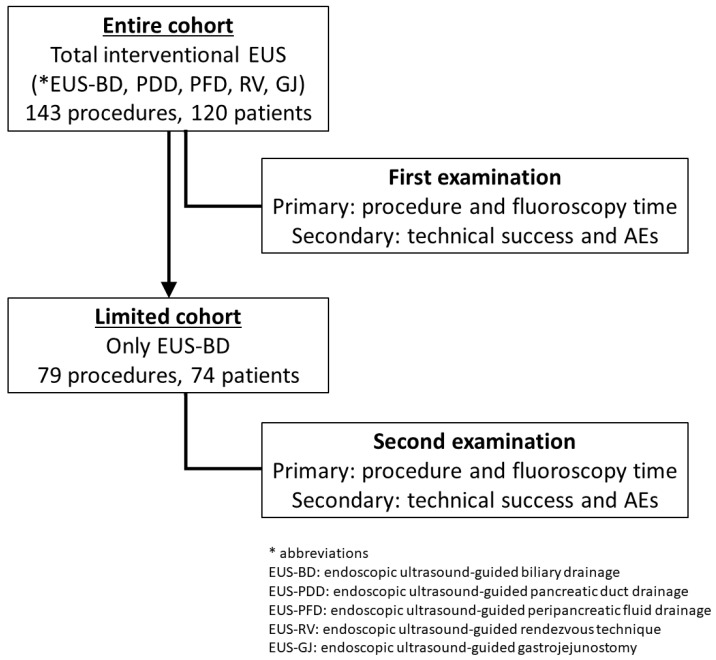
The flow chart of the patient selections and examinations regarding two cohorts: entire I-EUS and limited EUS-BD.

In total, 143 procedures in 120 patients were analyzed (Figure 2). The same or different procedures were performed more than once for different lesions in 22 patients. Of the 120 patients, the conventional EUS scope was used in 67 patients and the new EUS scope was used in 53 patients; none were treated with both. There were no significant differences in the patient characteristics between the two scopes, except the patient’s age. The patients treated with the new EUS scope were significantly older (conventional vs. new: 67 vs. 73; *p* = 0.048) (Table 2).

In total, 143 interventional EUS procedures was performed, including 84 EUS-BD, 13 EUS-PDD, 35 EUS-PFD, and 11 other procedures (Table 3). The conventional EUS scope was used in 82 procedures and the new EUS scope was used in 61 procedures. There were no differences between the two groups in terms of operator experience (expert vs. trainee; *p* = 0.497), procedure type (*p* = 0.803), puncture site (stomach vs. non-stomach; *p* = 0.984), stent type (metal vs. plastic; *p* = 0.247). As an analysis of the primary outcome, the new EUS scope had a significantly shorter procedure time (36.5 min, *p* = 0.026) than the conventional EUS scope (49 min). By contrast, the fluoroscopy time did not differ between the two groups (conventional vs. new: 14.5 min vs. 12.5 min; *p* = 0.269). As an analysis of the secondary outcome, there was no significant difference in the technical success between the two groups (conventional vs. new: 97.5% vs. 98.4%; *p* = 0.610). There were three unsuccessful cases, all of which occurred when EUS-HGS was attempted. In all of these cases, a GW could not be placed because of the insufficient dilation of the bile duct. Adverse events occurred in seven patients (8.5%) with the conventional EUS scope and three patients (4.9%) with the new EUS scope (*p* = 0.516).

### 5.2. Details of Adverse Events

There were nine adverse events, and except for one case of stent migration that occurred during EUS-PFD, all occurred during EUS-BD (Table 3). Adverse events occurred as early as within 14 days of the procedure. There were six cases in the conventional EUS scope group: two of biloma, one of biliary peritonitis, one of bleeding, one of pancreatitis (mild), and one of stent migration. The new scope had only one case each of biloma, biliary peritonitis, and bleeding. Stent migration during EUS-PFD occurred in the conventional EUS scope group. In a case in which the lumen-apposing metal stent (LAMS) migrated into the cyst at the time of stent placement, another LAMS was deployed, and the migrated LAMS was endoscopically retrieved through the second LAMS.

### 5.3. EUS-BD (Second Examination)

Regarding biliary drainage, 79 procedures were performed in 74 patients during the study period (Figure 2). Of the 74 patients, 43 were treated with the conventional EUS scope and 31 were treated with the new EUS scope; none were treated with both. Again, there were no significant differences in the patient’s characteristics between the two scopes except the patient’s age. The patients treated with the new EUS scope were significantly older (conventional vs. new: 66 vs. 73; *p* = 0.030) (Table 4).

In total, 79 EUS-BD procedures were performed, 47 and 32 with the conventional and new EUS scopes, respectively. There were no differences between the two groups in terms of operator experience, puncture site, or stent type (Table 5). According to the analysis of the primary outcome, the procedure time was significantly shorter for the new scope (50 min vs. 40 min, *p* = 0.020), as was the fluoroscopy time (18 min vs. 12.5 min, *p* = 0.044). According to the analysis of the secondary outcome, the technical success rate was high in both groups with no significant differences (conventional vs. new: 95.7% vs. 96.9%, *p* = 0.642). Adverse events occurred in six patients with the conventional EUS scope and three with the new EUS scope (*p* = 0.732).

### 5.4. Factors Associated with a Shorter Fluoroscopy Time

The median fluoroscopy time for the 79 procedures was 16 min, and we performed a subgroup analysis investigating the factors associated with a shorter fluoroscopy time between the groups with less than 16 min and more than 16 min of fluoroscopy times. Age and sex were excluded from the analysis because they were not considered to be related to fluoroscopy time (Table 6). Univariate analyses revealed that scope type (*p* = 0.035) and stent type (*p* = 0.019) differed significantly between the two groups. A multivariate analysis revealed that the new EUS scope (*p* = 0.046; odds ratio 0.368; 95% CI 0.138–0.984) and a metal stent (*p* = 0.025; odds ratio 0.322; 95% CI 0.120–0.869) were significantly associated with a shorter fluoroscopy time.

## 6. Discussion

We retrospectively compared the performances of a new EUS scope (EG-740UT) and a conventional EUS scope (EG-580UT). The new EUS scope significantly reduced the procedure time for entire I-EUS (entire cohort), and both the procedure time and fluoroscopy time for EUS-BD (limited cohort). A multivariate analysis revealed that the scope type and stent type were significantly associated with shortening fluoroscopy time.

### 6.1. Reduction in Radiation Exposure

Shortening the procedure time and fluoroscopy time reduces the burden on both the operator and the patient, but it is particularly important to shorten the fluoroscopy time [26,27,28]. The early and late effects of radiation exposure in normal tissues and organs are an issue for pancreatobiliary treatment, in which fluoroscopy is required [29]. In 2011 [30], the International Commission on Radiological Protection (ICRP) reduced the threshold dose for radiation-induced cataract from 150 millisievert (mSv) to 20 mSv per year, averaged over 5 years, with no single year exceeding 50 mSv to decrease the prevalence of cataract among healthcare workers [31]. The improved GW locking system of the new EUS scope prevents GW slippage and almost eliminates the need for exposure during device exchanges. As a result, the fluoroscopy time is significantly reduced with the new EUS scope. The stent type itself is unlikely to reduce the fluoroscopy time, and the double-GW technique for plastic stent placement may explain the increased fluoroscopy time. The double-GW technique requires a step to place the second GW, increasing the fluoroscopy time [32].

### 6.2. Procedure Time

The procedure time was significantly shorter using the new EUS scope for both the entire I-EUS and EUS-BD groups. The scanning area of the new EUS scope was wider than that of the conventional EUS scope, which reduces the blind area and prevents puncturing of blood vessels [33,34].

The new EUS scope has an enhanced forceps elevation and a reduced radius of curvature at the flexible tip. These improvements can allow for a greater selection of the puncture sites, and shorten the time from scope insertion to determining the puncture route. In addition, a tightened GW locking system might reduce the device exchange time, reducing the time from puncture to stent placement.

### 6.3. Procedure Safety

There were no significant differences between the two scopes in terms of adverse events. For the entire I-EUS group, the incidence rates of adverse events were 4.9% with the new EUS scope and 7.3% with the conventional EUS scope; for the EUS-BD only group, these incidence rates were 9.4% and 14.9%, respectively, which are lower than in the prior reports [35,36].

The new EUS scope has a CCD lens behind the working channel, enabling the endoscopist to recognize the stent during deployment. With the conventional EUS scope, the CCD lens is positioned in front of the working channel, and the angle and position of the scope must be significantly adjusted to visualize the stent. With the new EUS scope; however, the stent is readily visualized with only slight movement of the scope. We experienced a case of stent misplacement, in which a HOT AXIOS fell into a cyst during deployment [37,38]. This occurred with the conventional scope and could have been prevented by the new EUS scope.

### 6.4. Novelty of the Present Study

The new EUS scope, EG-740UT, is an echoendoscope developed exclusively for an interventional EUS, and this is the first study to examine its superiority over the conventional EUS scope in an interventional EUS. Recently, a single-arm study was reported to investigate the technical success rate of EUS-HGS using the EG-740UT [39]. This study reported a high technical success rate of 97.8%, similar to 96.9% in our study. Our study, however, includes not only EUS-BD but also the whole of Interventional EUS, and compares the superiority of a new EUS scope with a conventional EUS scope. Furthermore, our study differs from the previous report in that it included a substantial number of 143 procedures (45 procedures in the previous report) and that the primary outcome was the procedure time and the fluoroscopy time that directly affected the health of the patient and the physician.

## 7. Limitations

This study had several limitations. First, it was a retrospective study of a small number of cases. Second, it is unclear which improvements of the new scope are responsible for the reduced fluoroscopy time and procedure time. Third, the duration of each step of each procedure was not evaluated, so which steps were shortened is unknown. Fourth, the present study included only biliary and pancreatic drainage, peripancreatic fluid collection, and gastrojejunostomy, not vascular therapy, neurolysis for pain control, or drainage of the esophagus or pelvic organs. Fifth, the present study distinguishes between experts and trainees, but does not consider the potential skill and experience differences among operators.

## 8. Conclusions

The new EUS scope reduced the procedure time for the entire I-EUS group, and reduced both the procedure and the fluoroscopy time for EUS-BD compared to a conventional EUS scope.

## Figures and Tables

**Table 2:** Patient characteristics for the entire interventional EUS cohort.

Characteristic	Convention (EG-580UT) *n* = 67	New (EG-740UT) *n* = 53	*p*-Value
Sex (male/female)	44/23	31/22	0.420
Age *	67 (56–76)	73 (63–77)	0.048
Body mass index	19.3 (17.0–22.0)	20.6 (17.8–23.1)	0.592
Cause (malignancy/benign)	36/31	28/25	0.922
Performance status (0/1/2/3/4)	17/27/13/8/2	13/19/8/10/3	0.749
Charlson comorbidity index *	6 (1.0–7.0)	3 (0–7.0)	0.431
Antithrombotic drug	13 (19.4%)	14 (26.4%)	0.361
Systemic chemotherapy	19 (28.3%)	11 (20.7%)	0.339
Ascites	15 (22.3%)	10 (18.8%)	0.637
Prothrombin time (ratio) *	1.14 (1.05–1.23)	1.16 (1.07–1.28)	0.386
Platelet * (×10^4^/mL)	24.5 (15.1–33.4)	23.7 (16.4–33.3)	0.943
Total bilirubin * (mg/dL)	1.51 (0.81–7.17)	1.09 (0.74–7.45)	0.735

* Data are shown as the median (interquartile range).

**Table 3:** Results for the entire interventional EUS cohort.

Results	Convention (EG-580UT) *n* = 82	New (EG-740UT) *n* = 61	*p*-Value
Operator (expert/trainee)	41/41	34/27	0.497
Procedure * (BD/PDD/PFD/others) **	51/7/18/6	33/6/17/5	0.803
Puncture site (stomach/others)	70/12	52/9	0.984
Stent type (metallic/plastic)	42/35	38/21	0.247
Technical success	80 (97.5%)	60 (98.4%)	0.610
Procedure time * (min)	49 (32–62)	36.5 (25–57)	0.026
Fluoroscopy time * (min)	14.5 (9–23)	12.5 (9–19)	0.269
Adverse events	6 (7.3%)	3 (4.9%)	0.559
Biloma	2 (2.4%)	1 (1.6%)	0.610
Biliary peritonitis	1 (1.2%)	1 (1.6%)	0.673
Bleeding	1 (1.2%)	1 (1.6%)	0.673
Pancreatitis	1 (1.2%)	0	0.573
Stent migration	1 (1.2%)	0	0.573

* The procedure and fluoroscopy times are shown as the median (interquartile range); ** BD: biliary drainage, PDD: pancreatic duct drainage, PFD: peripancreatic fluid drainage.

**Table 4:** Patient characteristics for the limited EUS-BD cohort.

Characteristic	Convention (EG-580UT) *n* = 43	New (EG-740UT) *n* = 31	*p* Value
Sex (male/female)	30/13	15/16	0.063
Age *	66 (57–71)	73 (65–79)	0.030
Body mass index	21.8 (18.3–23.8)	19.3 (17.0–22.0)	0.275
Cause (malignancy/benign)	30/13	22/9	0.911
Performance status (0/1/2/3/4)	8/20/8/5/2	4/10/5/10/2	0.266
Charlson comorbidity index *	6 (3.5–7.0)	6 (1.5–8.0)	0.608
Antithrombotic drug	7 (16.2%)	7 (22.5%)	0.495
Systemic chemotherapy	13 (30.2%)	7 (22.5%)	0.465
Ascites	13 (30.2%)	7 (22.5%)	0.465
Prothrombin time (ratio) *	1.13 (1.02–1.22)	1.13 (1.05–1.22)	0.817
Platelet * (×10^4^/mL)	21.6 (13.1–33.5)	21.8 (15.1–33.1)	1.000
Total bilirubin * (mg/dL)	4.23 (1.47–8.95)	4.50 (0.98–9.87)	0.843

* Data are shown as the median (interquartile range).

**Table 5:** Results of the procedures for the limited EUS-BD cohort.

Results	Convention (EG-580UT) *n* = 47	New (EG-740UT) *n* = 32	*p*-Value
Operator (expert/trainee)	20/27	12/20	0.653
Puncture site (stomach/ others)	43/4	27/5	0.266
Technical success	45 (95.7%)	31 (96.9%)	0.642
Stent type (metallic/plastic)	27/18	20/11	0.690
Procedure time * (min)	50 (42–62)	40 (26–56)	0.020
Fluoroscopy time * (min)	18 (11–26)	12.5 (9–20)	0.044
Adverse events	5 (10.6%)	3 (9.3%)	0.855
Biloma	2 (4.2%)	1 (3.1%)	0.642
Biliary peritonitis	1 (2.1%)	1 (3.1%)	0.649
Bleeding	1 (2.1%)	1 (3.1%)	0.649
Mild pancreatitis	1 (2.1%)	0	0.649

* The procedure and fluoroscopy time are shown as the median (interquartile range).

**Table 6:** Analysis of the related factors for the fluoroscopy time.

Factor	<16 min (*n* = 38)	≥16 min (*n* = 41)	Univariate *p*-Value	Multivariate *p*-Value	Odds Ratio (95%CI *)
Operator (expert/trainee)	11/27	20/21	0.071	0.174	
Scope (conventional/new)	18/20	29/12	0.035	0.046	0.368 (0.138–0.984)
Etiology (malignant/benign)	29/9	26/15	0.097	0.649	
Puncture site (stomach/others)	32/6	38/3	0.236	0.094	
Plastic/metallic stent	10/28	20/18	0.019	0.025	0.322 (0.120–0.869)
Technical success (yes/no)	38/0	38/3	0.241	0.555	
Adverse events (yes/no)	2/38	6/35	0.168	0.238	

* CI: confidence interval.

## Data Availability

The data sets used and/or analyzed during the current study are available from the corresponding authors on reasonable request. However, the data sets include personal information. Therefore, limited information without personal information is available.
